# ETA-mediated anti-TNF-α therapy ameliorates the phenotype of PCOS model induced by letrozole

**DOI:** 10.1371/journal.pone.0217495

**Published:** 2019-06-06

**Authors:** Qin Lang, Xie Yidong, Zhang Xueguang, Wu Sixian, Xu Wenming, Zuo Tao

**Affiliations:** 1 Reproductive Medical Center, Department of Obstetrics and Gynecology, West China Second University Hospital, Sichuan University, Chengdu, Sichuan, China; 2 Sichuan University–The Chinese University of Hong Kong Joint Laboratory for Reproductive Medicine, West China Second University Hospital, Sichuan University, Chengdu, Sichuan, China; 3 Key Laboratory of Birth Defects and Related Diseases of Women and Children of Ministry of Education, West China Second University Hospital, Sichuan University, Chengdu, Sichuan, China; 4 West China School of Pharmacy, Sichuan University, Chengdu, Sichuan, China; Peking University Third Hospital, CHINA

## Abstract

Chronic inflammation is a typical characteristic of polycystic ovary syndrome (PCOS), in which, tumor necrosis factor (TNF)-α plays an important role. We investigated whether anti-TNF-α therapy can alleviate the core phenotypes of PCOS. In pubertal female Wistar rats, release pellets of letrozole (LET) were administered continuously for 90 days to induce PCOS-like phenotypes, followed by treatment with etanercept (ETA), a TNF-α inhibitor. ETA significantly inhibited increases in body weight and androgen, TNF-α, and MCP-1 levels, excessive recruitment of lipid droplets, altered levels of pre-adipose differentiation markers, and abnormal development of follicles. In addition, TNF-α and testosterone (T) levels in the rat sera were significantly positively correlated. Further experiments were performed to investigate the relationship between TNF-α and androgen. Persistent exposure of the RAW 264.7 cell line to low doses of testosterone significantly enhanced TNF-α expression and activated the NF-κB signaling pathway, which were blocked by ETA. Furthermore, treatment with TNF-α promoted the production of testosterone in KGN granulosa cells by reducing CYP19A1 expression, whereas ETA treatment blocked this process. In conclusion, anti-TNF-α therapy with ETA may be an efficient method to alleviate PCOS, whose underlying mechanism may be associated with its ability to reduce excessive androgen levels.

## Introduction

Polycystic ovary syndrome (PCOS) is a common endocrine disease that affects 6–21% women of reproductive age, approximately 75% of whom experience infertility due to anovulation [1,2]. Its pathological characteristics present diversity and heterogeneity, and include menstrual sparse or amenorrhea, chronic ovulation problem, infertility, increased hair growth, and acne, along with complications such as obesity, hyperandrogenism, hyperinsulinism, and chronic inflammation [3,4]. Although androgen plays an important role in the growth and development of follicles [5,6], excess androgen leads to a polycystic morphology of the ovary [7,8]. Furthermore, in previous epidemiological analyses, androgen levels were found to be associated with levels of other PCOS-related biomarkers, which reflected the characteristics of PCOS [9,10]. Hyperandrogenism has been regarded as a key causative factor of PCOS, and is widely accepted as one of the three core features of “Rotterdam Consensus Criteria,” which was the first international diagnostic standard of PCOS established in 2003 [11].

Chronic inflammation is considered as an important contributor to the pathogenesis of PCOS [12,13], which is also reflected by altered levels of inflammatory factors and their strong correlation with biomarkers of other PCOS phenotypes [14–16],. Tumor necrosis factor alpha (TNF-α), one of the well-known inflammatory factors, was demonstrated as a potential mediator of the PCOS-related physiological processes such as obesity, insulin resistance, and androgen expression [15,17,18]. Further, both excessive androgen levels and chronic inflammation play important roles in the pathogenesis of PCOS, and formed a complex interactive network with other factors. Therefore, it would be interesting to elucidate whether anti-inflammatory therapy could significantly alleviate the abnormal symptoms of PCOS.

Several chemicals, most of which are androgen and its derivatives, have been used in mouse and rat to induce phenotypes that mimic those of PCOS [19,20]. However, recently, letrozole, a nonsteroidal aromatase inhibitor that elicits more significant and comprehensive phenotypes of PCOS, has been regarded as a better PCOS inducer than other agents [21,22]. ETA, a fusion protein of the TNF receptor and IgG1 Fc that exhibits less potential safety risk compared to other inhibitors, was the first TNF-α inhibitor to be approved for clinical use in rheumatic diseases [23,24].

The direct relationship between androgen and inflammatory factors in PCOS has not been elucidated yet. In some studies, androgen seemed to be an efficient protective agent against the negative influences of TNF-α [25,26], which was not consistent with the conclusions in PCOS. On the other hand, inflammation was demonstrated to influence androgen signaling thereby regulating androgen-responsive proteins [27,28]. Some other studies have focused on the regulatory role of inflammation or TNF-α in androgen expression. Therefore, in this study, we designed two *in vitro* experiments on mouse macrophage cells (RAW 264.7 cell line) and ovarian granulosa cells (KGN cell line) to investigate the direct association between androgen and TNF-α, and explore the potential underlying mechanisms. Furthermore, ETA was employed to attenuate the possible abnormal alterations induced by androgen or TNF-α.

Since chronic inflammation is a key contributor to the pathogenesis of PCOS, the current study applied anti-TNF-α therapy using ETA in a letrozole-induced PCOS rat model. In addition, direct interaction between TNF-α and androgen and their roles in physiological activities were also investigated.

## Materials and methods

### Animal model of PCOS

**Fig 1:**
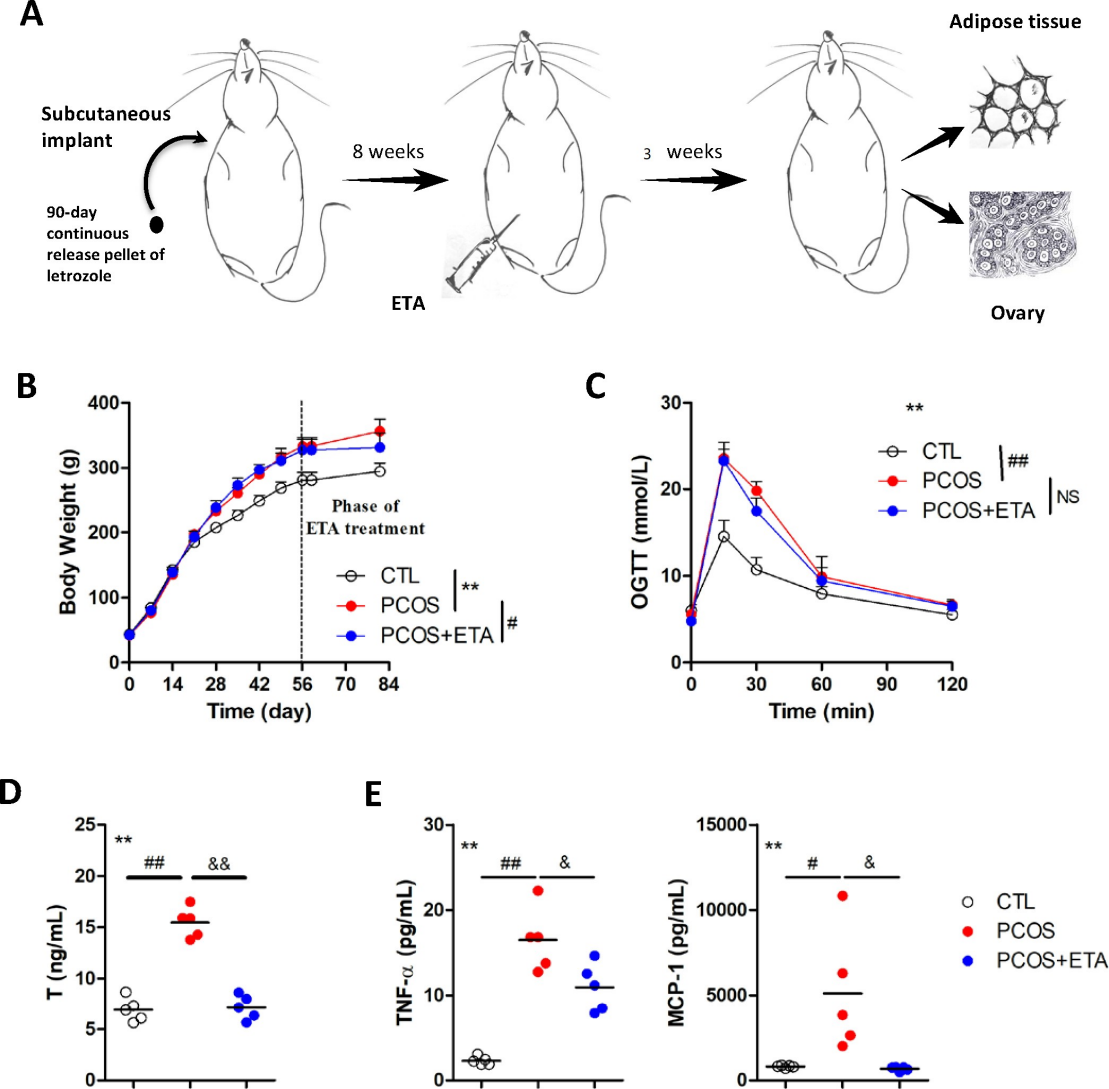
Immunological therapy of polycystic ovary syndrome (PCOS) rat. (A) PCOS model of rat was induced by subcutaneously implanted 90-day continuous release pellet of letrozole for 8 weeks, followed by 3 weeks of immunological therapy of etanercept (ETA, a TNF-α antagonist). Sera, adipose tissues, and the ovaries of the rats were harvested at the end of the treatment. ETA significantly attenuated the (B) increased weight (**CTL versus PCOS, *P*<0.01 by repeated measure ANOVA; ^#^PCOS versus PCOS+ETA, *P*<0.05 by t-test), (D) serum levels of testosterone (**All groups, *P*<0.01 by one-way ANOVA; ^##^CTL versus PCOS, *P*<0.01 by Dunnett’s multiple comparison test; ^&&^PCOS versus PCOS+ETA, *P*<0.01 by Dunnett’s multiple comparison test), and other inflammatory factors, including (E) TNF-α and MCP-1 (**All groups, *P*<0.01 by one-way ANOVA; CTL versus PCOS, ^#^*P*<0.05 and ^##^*P*<0.01 by Dunnett’s multiple comparison test; ^&^PCOS versus PCOS+ETA, ^&^*P*<0.05 and ^&&^*P*<0.01 by Dunnett’s multiple comparison test) in the PCOS rats. (C) The increased glucose tolerance level did not change significantly (**All groups, *P*<0.01 by repeated measure ANOVA; ^##^CTL versus PCOS, *P*<0.01 by Dunnett’s multiple comparison test; ^NS^PCOS versus PCOS+ETA, *P*>0.05 by Dunnett’s multiple comparison test).

Fifteen female Wistar rats aged 21 d were purchased from Dashuo Experimental Animal Co. Ltd. (Chengdu, China), and housed (five rats per cage) under controlled conditions (25°C, 12 h light/day). The study was approved by the Animal Ethics Committee of Sichuan University (grant number: WCSUH-2018-32). The rats were implanted subcutaneously with 90-day continuous-release pellets (Novartis Pharma AG, Basel, Switzerland). The pellets for polycystic ovary syndrome (PCOS) model rats released 200 μg letrozole (LET) per day, while the control pellets lacked the bioactive molecule. The PCOS model rats were treated with experimental drug etanercept (Enbrel, ETA, 5 mg/kg per 4 d) 8 weeks post implantations. All the rats were sacrificed after 3 weeks of ETA treatment (Fig 1A). In the above operations, intraperitoneal injection of pentobarbital sodium was applied to alleviate the sufferings of the rats.

### Tests for body weight, androgen level, and oral glucose tolerance

Rats were weighed weekly on electronic scales during induction of polycystic ovary syndrome (PCOS), before the beginning of the treatments with the medicines, and at the end of the treatments. Testosterone (T) level in the rat serum, measured by GC-MS before and after the treatments, was used to represent the androgen levels. On the final day, the rats were fasted overnight before oral administration of glucose (2 mg/kg body weight), and blood glucose levels were measured using ACCU-CHEK (Roche, Switzerland) at 0, 15, 30, 60, and 120 min post-gavages.

### Measurement of multi-inflammatory factors

Multi-inflammatory factors in rat serum, including TNF-α, MCP-1, IFN-γ, IL-1α, IL-1β, IL-2, IL-6, IL-10, and IL-17, were analyzed using Merck Millipore suspension array kit. The inflammatory factors secreted by RAW 264.7 cell line induced by lipopolysaccharide (LPS) were also analyzed using the same suspension array kit, and TNF-α induced by testosterone (T) was determined using ELISA kit (Neobioscience, Shenzhen, China).

### Cell culture

RAW 264.7 and KGN cell lines were provided by Dr. Ma Yaxian (West China Second University Hospital), and Dr. Wang Fei (Chengdu Institute of Biology, Chinese Academy of Sciences), respectively, who purchased them from ATCC. RAW 264.7 cells were maintained in DMEM medium (Gibco, New York, USA) supplemented with 10% FBS (Gibco, New York, USA), while KGN cells in DMEM/F12 medium (Gibco, New York, USA) supplemented with 10% FBS. Both of them were incubated at 37°C in a humidified atmosphere with 5% CO_2_. RAW 264.7 cells were treated with different doses of testosterone (0, 10, and 100 nM) for 3 d. Etanercept (ETA) was used to treat the inflammation of RAW 264.7 cells induced by testosterone (100 nM) and LPS (1 μg/mL).

### H&E staining and immunofluorescence

After overnight fixing with 4% PFA, fresh tissue samples were dehydrated by gradually exposing to 50%, 70%, 80%, 90%, and 100% (twice) ethanol, and dimethylbenzene (twice) at room temperature. Then, they were embedded with paraffin at 60°C and cut into sections. Paraffin sections were dewaxed by following the reversed steps of embedment, and then washed twice with distilled water before staining. The sections were stained with hematoxylin for 5 min, treated with the differentiation fluid (1% hydrochloric acid alcohol) for 30 sec, washed with tap-water for 15 min, and then stained with eosin for 2 min. Lastly, the stained sections were dehydrated, and sealed with coverslips and imaged under microscope(Olympus, Japan).Before immunofluorescence, sections were incubated in boiling 0.01 M citrate buffer for 20 min to fix the antigens after dehydration. Cell slides were then fixed by overnight incubation with 4% PFA. Subsequently, the samples were sealed with 3% BSA, incubated overnight at 4°C with primary antibodies, followed by incubation with fluorescent secondary antibodies, and then with DAPI, and finalized. The primary antibody for P65 (1:100 dilution) was purchased from Proteintech Group (Chicago, USA) and for CYP19A1 (1:100 dilution) from Boster Biotech Co. Ltd. (Wuhan, China).

### Reverse transcription and real time polymerase chain reaction

Total RNA was extracted using Trizol (Life Tech, Invitrogen, California, USA), according to the manufacturer’s instructions, and the concentration was measured using Nanodrop 2000 (Thermo, MA, USA). The RNA was reverse transcribed using reverse transcription kit (Takara, Japan) and a Thermal cycler (Bio-rad, California, USA), and real-time qPCR was performed using the SYBR Green system (Life Tech, Invitrogen, USA) in an ABI 7500 (Life Tech, Invitrogen, California, USA), as per the manufacturer’s instructions. Expression of target genes was measured using the 2^-ΔΔCt^ method, and actin was used as the exogenous control gene. The primers were designed using Primer Premier 5.0 (Premier, Canada) and synthesized by Chengdu Diqiao Biotechnology Co., Ltd. (Chengdu, China).

### Western blot analysis

To obtain the protein, tissue samples and cells were lysed using RIPA lysis buffer and centrifuged at 12,000×g for 20 min to collect the supernatant without lipid. Protein concentration was measured using the BCA protein assay (Thermo Scientific, MA, USA). Equal amounts of protein were loaded onto an SDS-polyacrylamide gel, electrophoresed, and transferred to polyvinylidene difluoride membranes (PVDF, Minipore, MA, USA). The membranes were blocked with 5% non-fat milk or bovine serum albumin (BSA) at room temperature for 1 h, and then incubated with primary antibodies at 4°C for overnight. Each membrane was washed with TBST three times for 15 min followed by incubation with an HRP-conjugated secondary antibody (Zhongshan Jiangqiao, Beijing, China) at room temperature for 1 h. Finally, each membrane was developed using an enhanced chemiluminescence (ECL) detection kit (Minipore, MA, USA) and visualized using X-OMAT BT film (Carestream, Toronto, Canada). The antibodies for TNF-α, TNFR1, and TNFR2 were purchased from Santa Cruz (California, USA), the primary antibodies for AR, IκB, NF-κB, and P65 from Proteintech Group (Chicago, USA), TNF-α from CST (Boston, USA), and β-actin, and GAPDH from Zenbio Biotech Co., Ltd. (Chengdu, China).

### Enzyme linked immunosorbent assay (ELISA)

TNF-α ELISA kits for both mouse and rat species were purchased from Neobioscience Biotech Co. Ltd. (Shenzhen, China). Testosterone ELISA kits for rat and human were obtained from Cusabio (Wuhan, China). The levels of TNF-α and testosterone were measured according to the manufacturers’ instructions.

### Statistical analysis

The data are presented as mean ± SD. Two groups were compared by Student’s *t*-test, whereas, more than two groups were compared by One-way ANOVA, in which each pair was evaluated by post-hoc analysis. Statistical significance was set at P<0.05.

## Results

### PCOS model and etanercept therapy

The PCOS model of rat was established by implanting continuous-release letrozole pellets for 8 weeks, and subsequent ETA therapy for 3 weeks (Fig 1A). Overweight (Fig 1B), higher glucose tolerance (OGTT, Fig 1C), elevated serum testosterone levels (T, Fig 1D), and excessive TNF-α and MCP-1 levels (Fig 1E) were observed after the model establishment, of which, all the altered parameters, except OGTT, were significantly reduced after the ETA therapy (Fig 1B–1E). It indicated successful establishment of the PCOS model, which mimicked the core clinical phenotypes of PCOS patients, which were partially alleviated by ETA.

### Adipose tissue and ovarian conditions after ETA treatment

**Fig 2:**
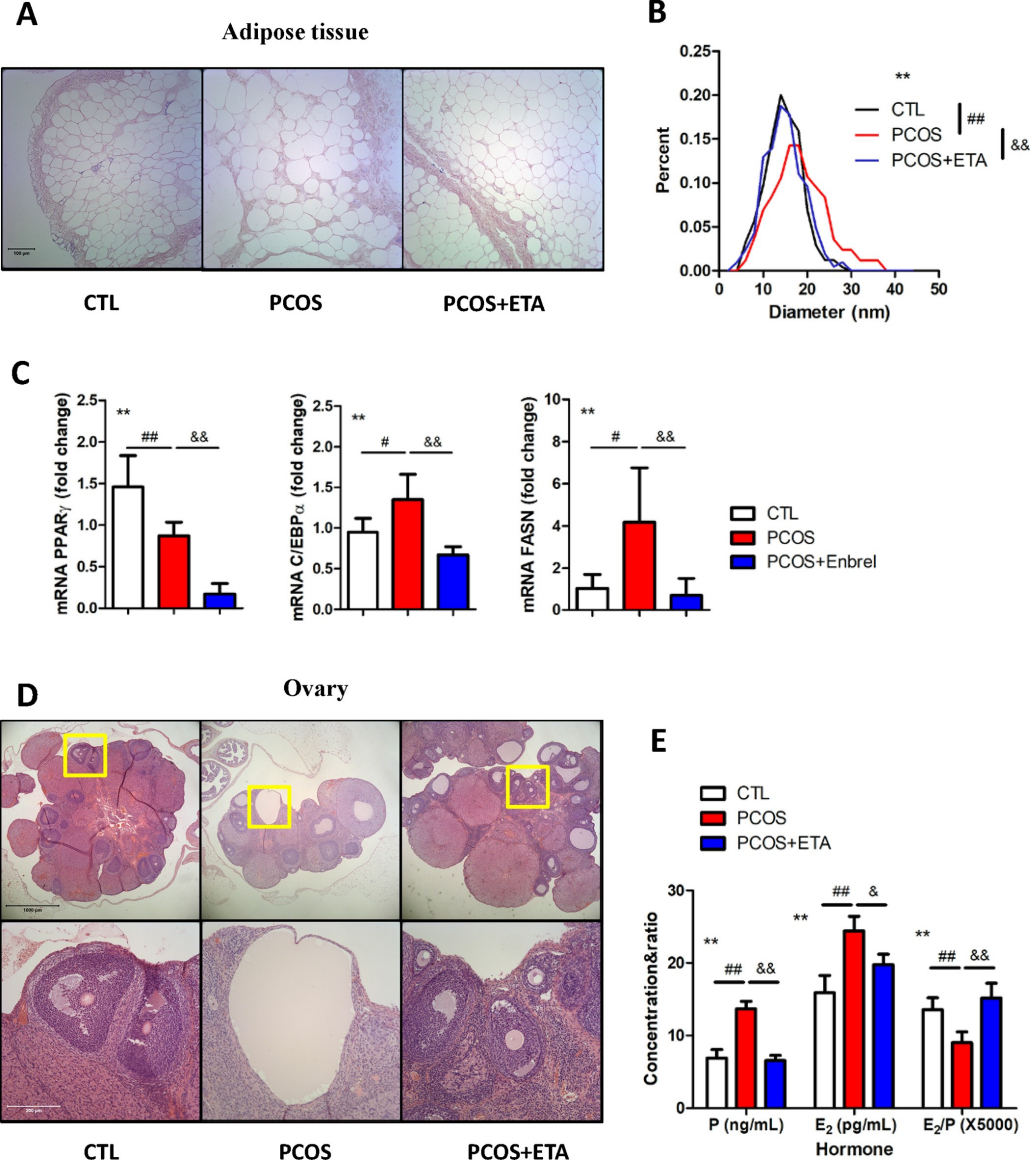
Effects of etanercept (ETA) on adipose tissue and ovary. ETA therapy significantly alleviated the obesity of PCOS rat by (A) decreasing the elevated size of the adipocytes and (B) restoring the size distribution similar to normal adipocytes in PCOS rats (**cell sizes of all groups, *P*<0.01 by one-way ANOVA; ^##^CTL versus PCOS, *P*<0.01 by Dunnett’s multiple comparison test; ^&&^PCOS versus PCOS+ETA, *P*<0.01 by Dunnett’s multiple comparison test). (C) ETA significantly inhibited the mRNA expressions of PPARγ, C/EBPα, and FASN (**All groups, *P*<0.01 by one-way ANOVA; CTL versus PCOS, ^#^*P*<0.05 and ^##^*P*<0.01 by Dunnett’s multiple comparison test; ^&&^PCOS versus PCOS+ETA, *P*<0.01 by Dunnett’s multiple comparison test), indicating the adverse effects on the differentiation and lipid recruitment of adipocyte in PCOS rat. (D) ETA also alleviated the abnormal morphology of the ovary of PCOS rat. (E) The levels of estradiol (E_2_) and progesterone (P) were significantly inhibited after ETA treatment, while the ratio of E_2_/P was significantly increased in the ETA-treated group (**All groups, *P*<0.01 by one-way ANOVA; ^##^CTL versus PCOS, *P*<0.01 by Dunnett’s multiple comparison test; PCOS versus PCOS+ETA, ^&^*P*<0.05 and ^&&^*P*<0.01 by Dunnett’s multiple comparison test).

Obesity and chronic ovulation are often considered as clinical manifestations of PCOS. In fact, our model rats exhibited larger adipose vacuoles, whose diameter was significantly decreased by ETA treatment (Fig 2A and 2B). Furthermore, ETA also inhibited the mRNA expression of the markers associated with adipose differentiation and lipid recruitment, including PPARγ, C/EBPα, and FASN, among which, the expression of C/EBPα and FASN increased in the PCOS, whereas the expression of PPARγ decreased (Fig 2C). In addition, H&E staining of the ovary revealed that letrozole (LET) promoted the abnormal growth of follicles, in which no oocyte exists, whereas ETA treatment significantly reduced the growth rate of these follicles (Fig 2D). Furthermore, the increased progesterone/E_2_ levels, which were reported to be tightly associated with follicular development, induced by LET, were also attenuated after ETA treatment (Fig 2E). Together, the results indicated that LET induced the PCOS model with altered adipose tissue morphology and metabolism, which could be largely rescued by ETA treatment.

### Persistent exposure of androgen-induced inflammation through the TNF-α-NF-κB mediated pathway

**Fig 3:**
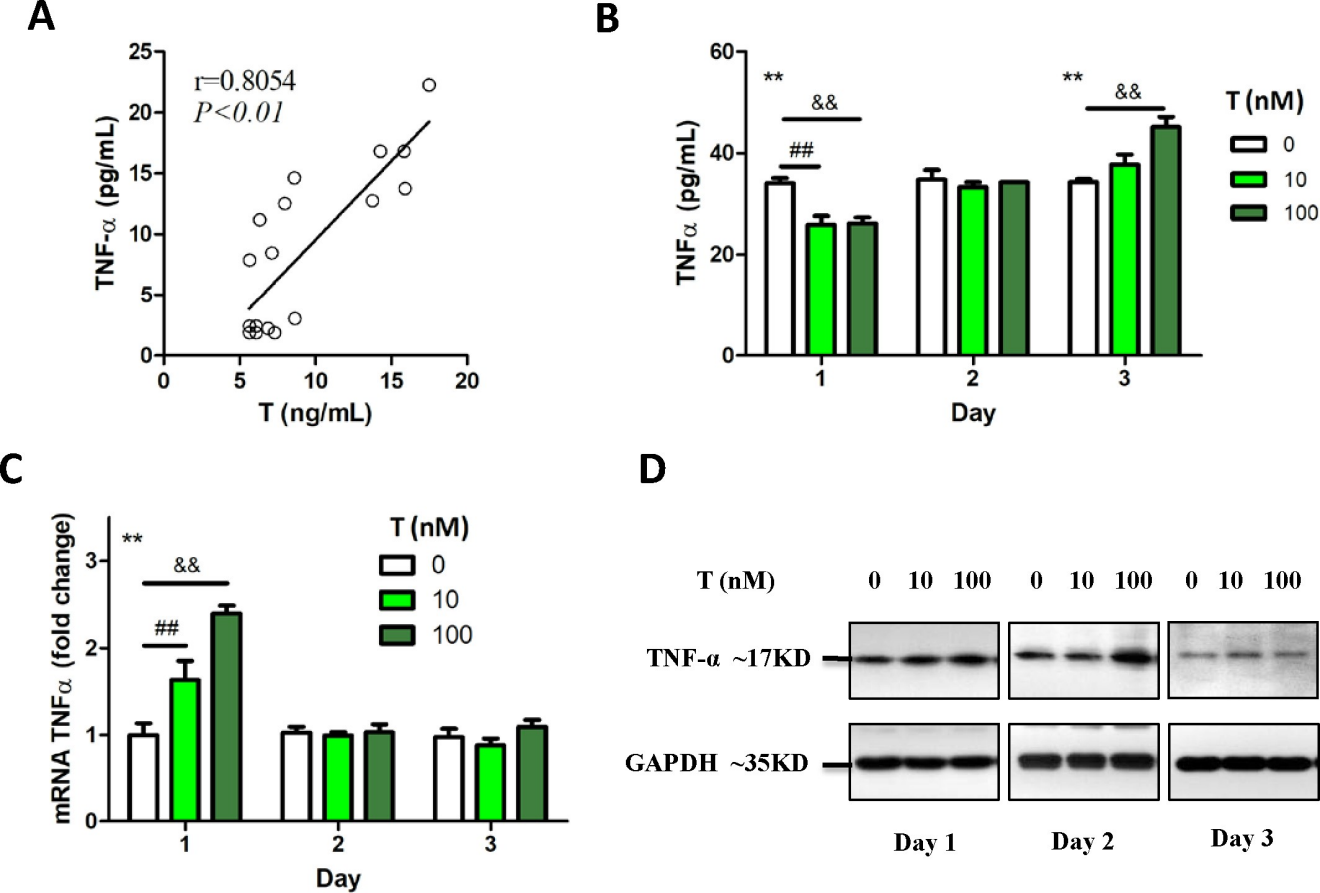
Influence of persistent testosterone (T) exposure on TNF-α secretion. (A) A significant positive correlation was found between serum T level and TNF-α level in the rats (r = 0.8054, *P*<0.01). (B) RAW 264.7 cells were persistently exposed to different doses of T (0, 10, and 100 nM). The level of TNF-α secreted into the medium decreased significantly at day-1. However, it increased significantly compared to the control at day-3, indicating the bi-directional regulation of T (**three dosages at the same time point, *P*<0.01 by one-way ANOVA; ^##^group treated with 0 nM T versus group treated with 10 nM T, *P*<0.01 by Dunnett’s multiple comparison test; ^&&^group treated with 0 nM T versus group treated with 100 nM T, *P*<0.01 by Dunnett’s multiple comparison test). (C) The expression of TNF-α mRNA increased after stimulation with T, which restored to the normal levels after day-1 (**three dosages at the same time point, *P*<0.01 by one-way ANOVA; ^##^group treated with 0 nM T versus group treated with 10 nM T, *P*<0.01 by Dunnett’s multiple comparison test; ^&&^group treated with 0 nM T versus group treated with 100 nM T, *P*<0.01 by Dunnett’s multiple comparison test). (D) The expression of TNF-α protein in the cells treated with T increased significantly at day-1 and day-2.

A significant positive correlation was found between the levels of TNF-α and testosterone (T) in rat sera (Fig 3A). Therefore, we used macrophage cells as a model to investigate the relationship between TNF-α and T *in vitro*. Mouse macrophage cells (RAW 264.7) were exposed to different doses of T (0, 10, and 100 nM) for 3 d. ELISA results showed that high level of T (100 nM) significantly increased the TNF-α levels at day 3 (Fig 3B). However, real-time PCR and western blot analysis indicated that T increased the expression of TNF-α only at day-1 post treatment, which was restored to the normal level upon persistent T exposure (Fig 3C and 3D). The later increase in levels of secreted TNF-α compared to the shorter response of TNF-α at mRNA and protein level indicated a possible transcriptional regulation of TNF-α by T.

**Fig 4:**
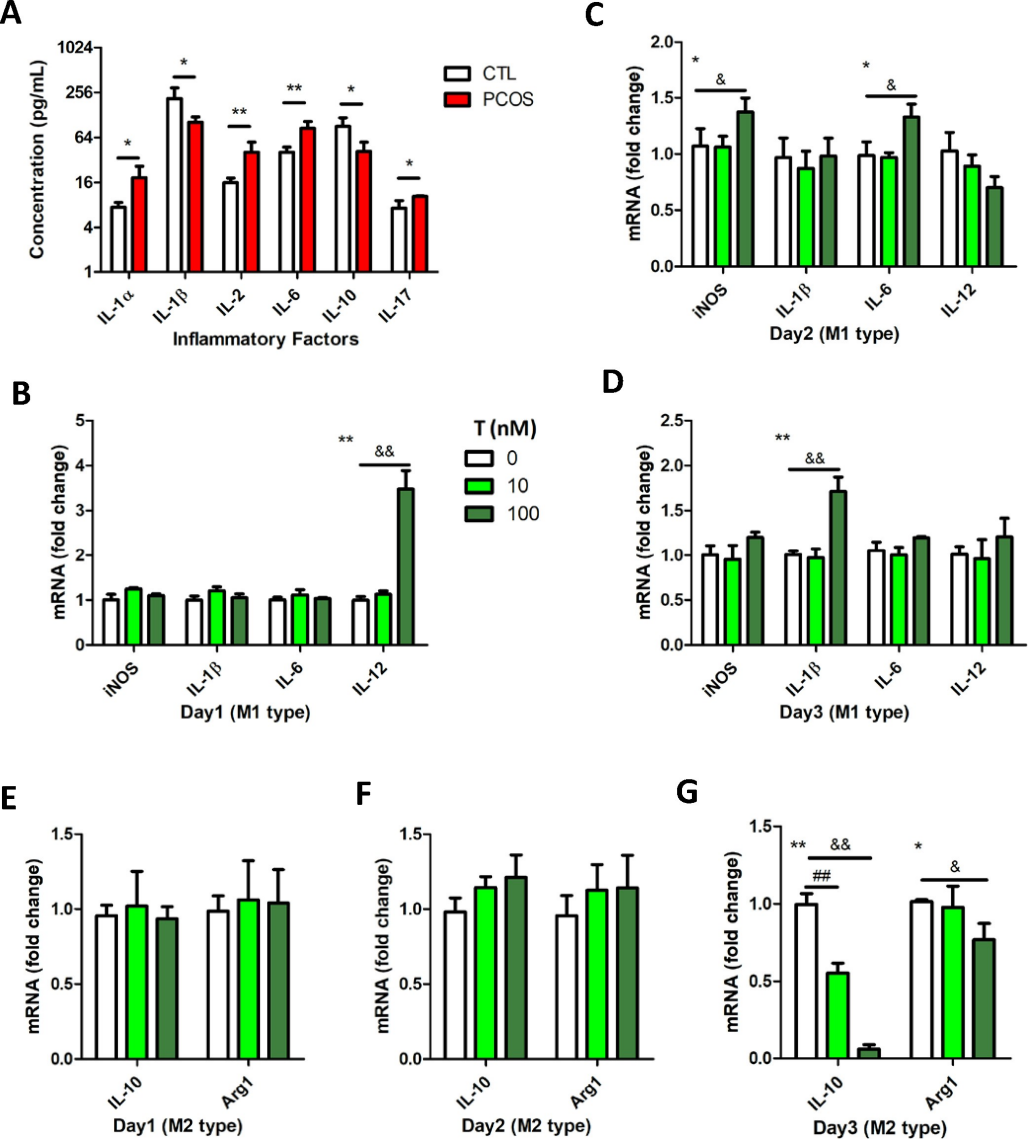
M1 polarization of macrophages upon persistent exposure to androgen. (A) Upon measurement, IL-1, IL-2, IL-6, and IL-17 cytokinesexhibited altered expressions in the PCOS rats. The serum levels of testosterone (T) and almost all the factors associated with M1 macrophages were increased significantly, whereas IL-10 level, which is associated with M2 macrophages, decreased (CTL versus PCOS, *P<0.05 and **P<0.01 by t-test). (B, C, and D) Upon stimulation of RAW 264.7 cells with T, the mRNA expression levels of IL-12, iNOS and IL-6, and IL-1β increased significantly on day-1, 2, and 3, respectively, whereas (E, F, G) IL-10 and Arg1 expressions decreased significantly on day-3 (treatment with three T doses at the same time point, *P<0.05 and **P<0.01 by one-way ANOVA; group treated with 0 nM T versus group treated with 10 nM T, ^#^*P*<0.05 and ^##^*P*<0.01 by Dunnett’s multiple comparison test; group treated with 0 nM T versus group treated with 100 nM T, ^&^*P*<0.05 and ^&&^*P*<0.01 by Dunnett’s multiple comparison test).

Cytokine profiling revealed the increased expression of certain inflammatory factors, including IL-1α, IL-2, and IL-6, and decrease in the levels of other cytokines in PCOS model (Fig 4A). Therefore, we examined whether T exposure could alter the cytokine expression in macrophage cell model. The result indicated that macrophage showed M2 type characteristic upon short-term T treatment, but was transformed to M1 type upon persistent exposure to T (Fig 4C–4G).

**Fig 5:**
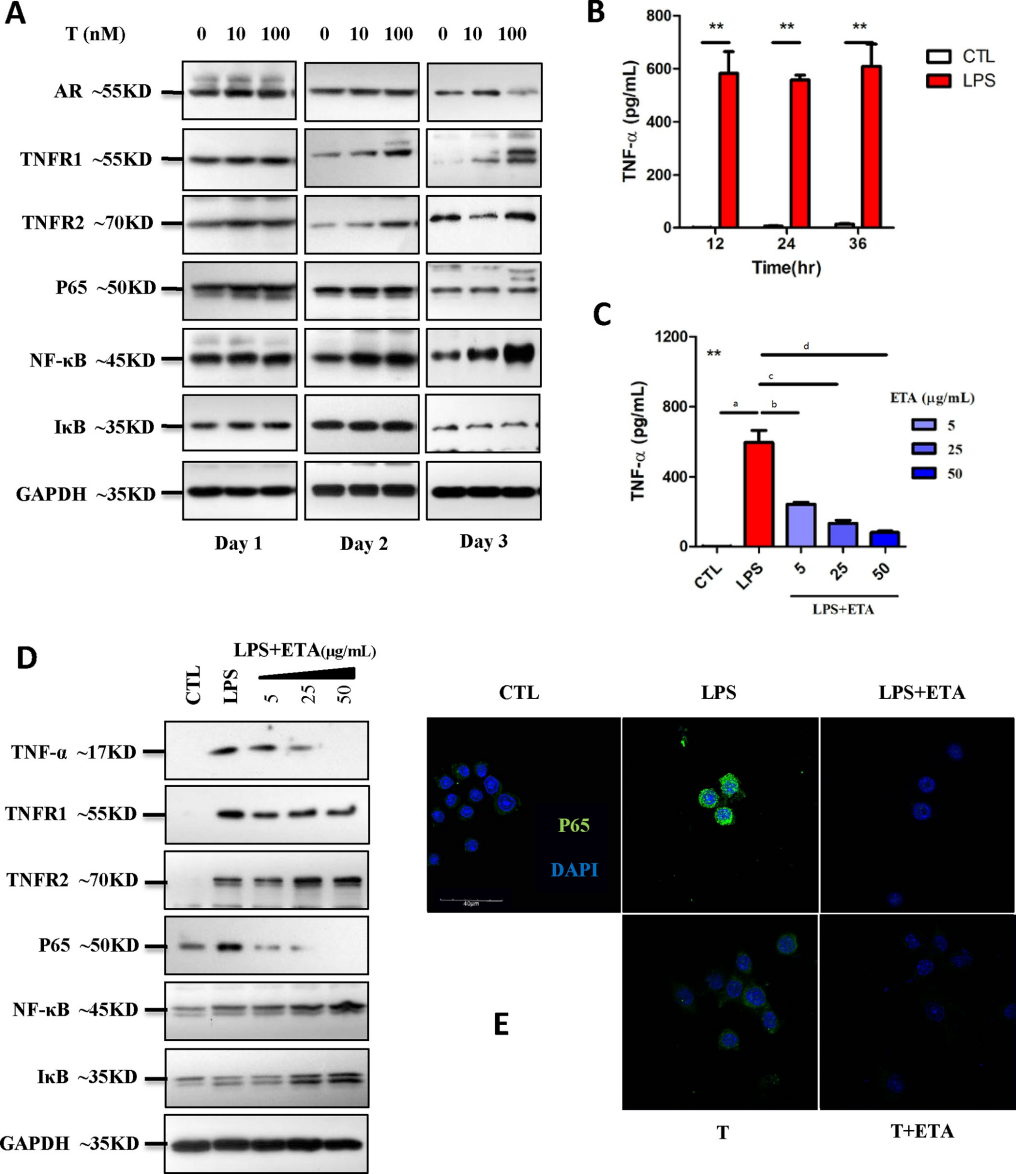
Etanercept (ETA) therapy altered NF-κB signaling pathway. (A) Upon stimulation of RAW 264.7 cells with the testosterone (0, 10, and 100 nM), the expressions of AR, TNFR1, and TNFR2 and the levels of secreted TNF-α, NF-κB, P65, and IκB increased significantly, indicating the activation of NF-κB signaling pathway. (B) Lipopolysaccharide (LPS, 1 μg/mL) stimulated RAW 264.7 cells to secrete TNF-α for 12, 24, and 36 h post treatment (**CTL versus LPS, *P*<0.01), (C) which was significantly inhibited by ETA (5, 25, and 50 μg/mL) (**All groups, *P*<0.01 by one-way ANOVA; a indicates CTL versus LPS, *P*<0.01 by Dunnett’s multiple comparison test; b,c,d indicate LPS versus LPS+ETA treatment group, *P*<0.01 by Dunnett’s multiple comparison test). Furthermore, (D) ETA significantly inhibited the LPS-induced expressions of TNF-α, TNFR1, and P65. (E) Immunostaining of P65 indicated that ETA inhibited the translocation of P65 into nucleus induced by LPS, indicating the capability of ETA to suppress the activated NF-κB signaling pathway.

TNF-α activates the NF-κB signaling pathway. Therefore, we examined the effect of T exposure on the downstream pathway and on the effect of ETA. Although short-term exposure (day-1) to T did not influence TNF-α levels or NF-κB signaling pathway significantly, persistent exposure significantly promoted expression of AR, TNF-α, TNFR1, and TNFR2 proteins, and activated NF-κB signaling pathway by enhancing the expressions of P65, NF-κB, and IκB proteins (Fig 5A). Lipopolysaccharide (LPS) was utilized to induce a classic inflammatory response in RAW 264.7 cells. ETA significantly inhibited the TNF-α secretion (Fig 5B and 5C). Furthermore, ETA significantly attenuated the LPS-induced overexpression of TNF-α, TNFR1, and P65 proteins (Fig 5D), indicating that ETA significantly suppressed the NF-κB signaling pathway. In addition, ETA inhibited the translocation of P65 from cytoplasm to nucleus induced by persistent T exposure as well as LPS treatment (Fig 5E). Thus, ETA attenuates the NF-κB-mediated inflammation response upon LPS treatment or T exposure.

### Influence of TNF-α on testosterone accumulation in ovary

**Fig 6:**
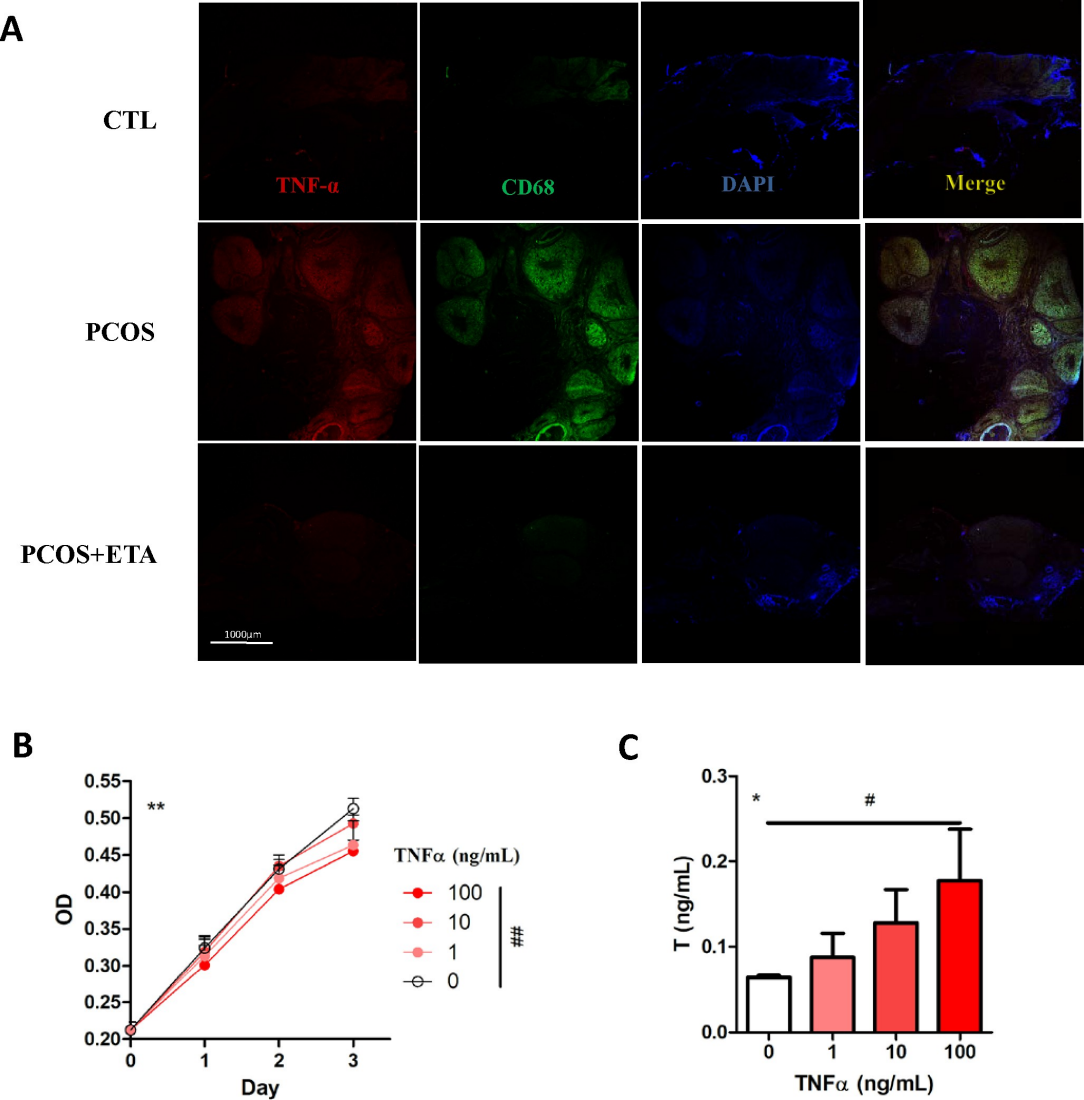
Influence of TNF-α on the secretion of testosterone (T). (A) Immunostaining revealed that the TNF-α expression was significantly increased in the ovary of PCOS rat compared to that of the control and decreased after the ETA therapy (CD68 was used as a macrophage marker). Upon using pregnenolone as substrate, (C) TNF-α-treated KGN cells secreted more T compared to the normal cells (**All groups, *P*<0.01 by repeated measure ANOVA; ^##^group treated with 0 ng/mL T versus group treated with 100 ng/mL T, *P*<0.01 by Dunnett’s multiple comparison test), although (B) TNF-α inhibits proliferation of the cells at day-3 (*All groups, *P*<0.05 by one-way ANOVA; ^#^group treated with 0 ng/mL T versus group treated with 100 ng/mL T, *P*<0.05 by Dunnett’s multiple comparison test).

**Fig 7:**
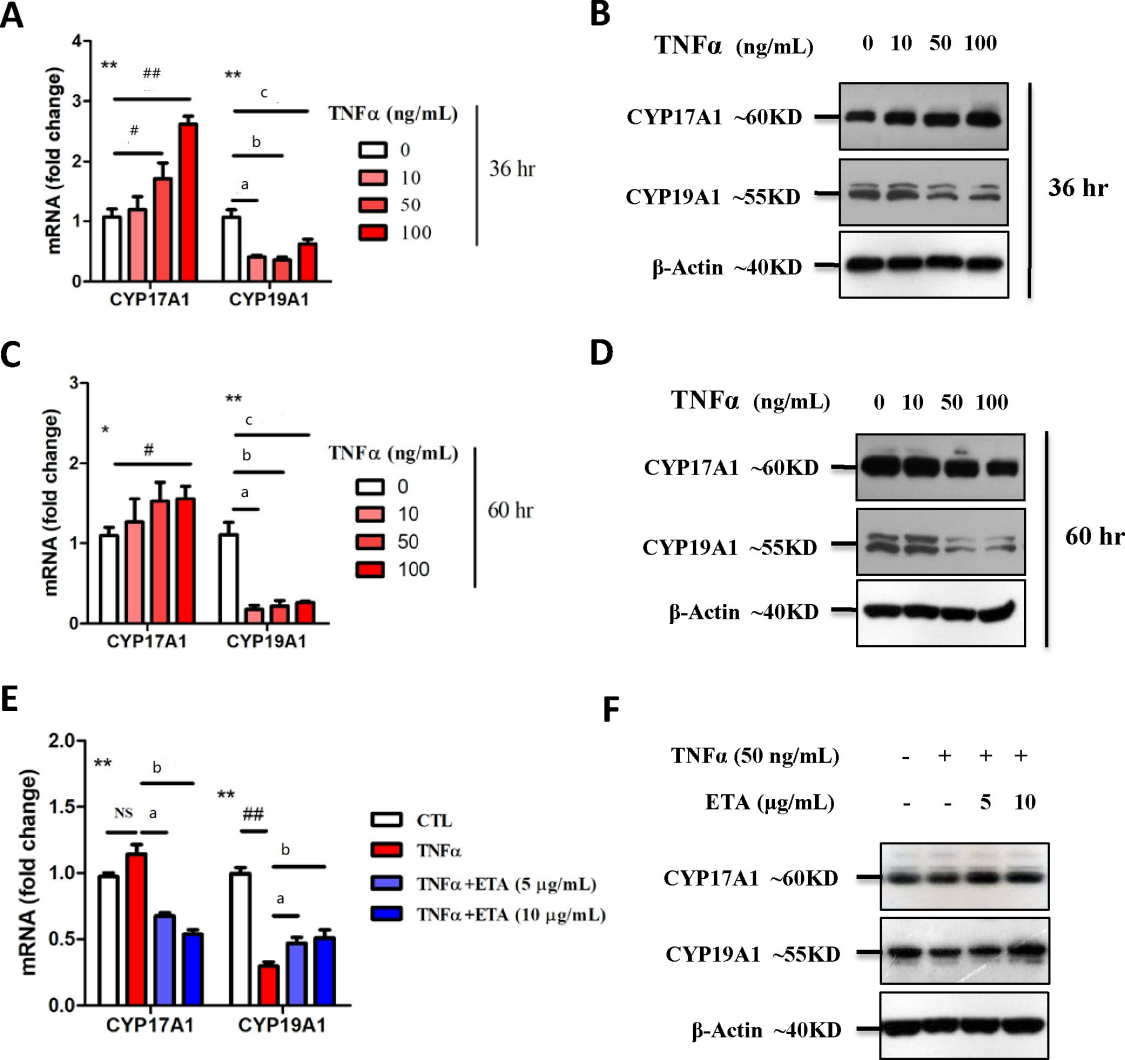
Regulation of TNF-α and etanercept (ETA) on the enzymes associated with hormone conversion. (A-D) The mRNA and protein expressions of CYP19A1 in KGN cells were significantly inhibited after 36 h of persistent treatment with different doses of TNF-α (a,b,c indicate comparison of control with 10, 50, and 100 ng/mL of ETA). However, the influence of TNF-α on CYP17A1 expression could not be determined due to the different expressions of CYP17A1 at different time points compared to that of the control (All groups, *P<0.05 and **P<0.01 by one-way ANOVA; CTL versus TNF-α, ^#^*P*<0.05 and ^##^*P*<0.01 by Dunnett’s multiple comparison test). (E, F) ETA ameliorated the downregulation of CYP19A1 induced by TNF-α. However, ETA-mediated regulation of CYP17A1 was still undetermined (All groups, *P<0.05 and **P<0.01 by one-way ANOVA; CTL versus TNF-α, ^#^*P*<0.05 and ^##^*P*<0.01 by Dunnett’s multiple comparison test; TNF-α versus TNF-α+ETA, ^&^*P*<0.05 and ^&&^*P*<0.01 by Dunnett’s multiple comparison test).

Excess TNF-α secretion has been considered to be a characteristic of PCOS [29]. Immunofluorescence staining showed excessive TNF-α staining in the ovary of PCOS model rats, which was inhibited by ETA treatment (Fig 6A). In KGN cells, significant growth inhibition and elevated T levels (10 μM pregnenolone as substrate) were observed after the stimulation of TNF-α (Fig 6B and 6C), suggesting that local production of testosterone (T) could also be affected by local TNF-α secretion. TNF-α also promoted CYP17A1 expression and significantly inhibited CYP19A1 expression at both mRNA and protein levels (Fig 7A–7D), which was significantly rescued by ETA (Fig 7E and 7F), indicating that the mechanism underlying the TNF-α-mediated regulation of T production could be associated with the regulation of cytochrome P450 expression in ovary.

**Fig 8:**
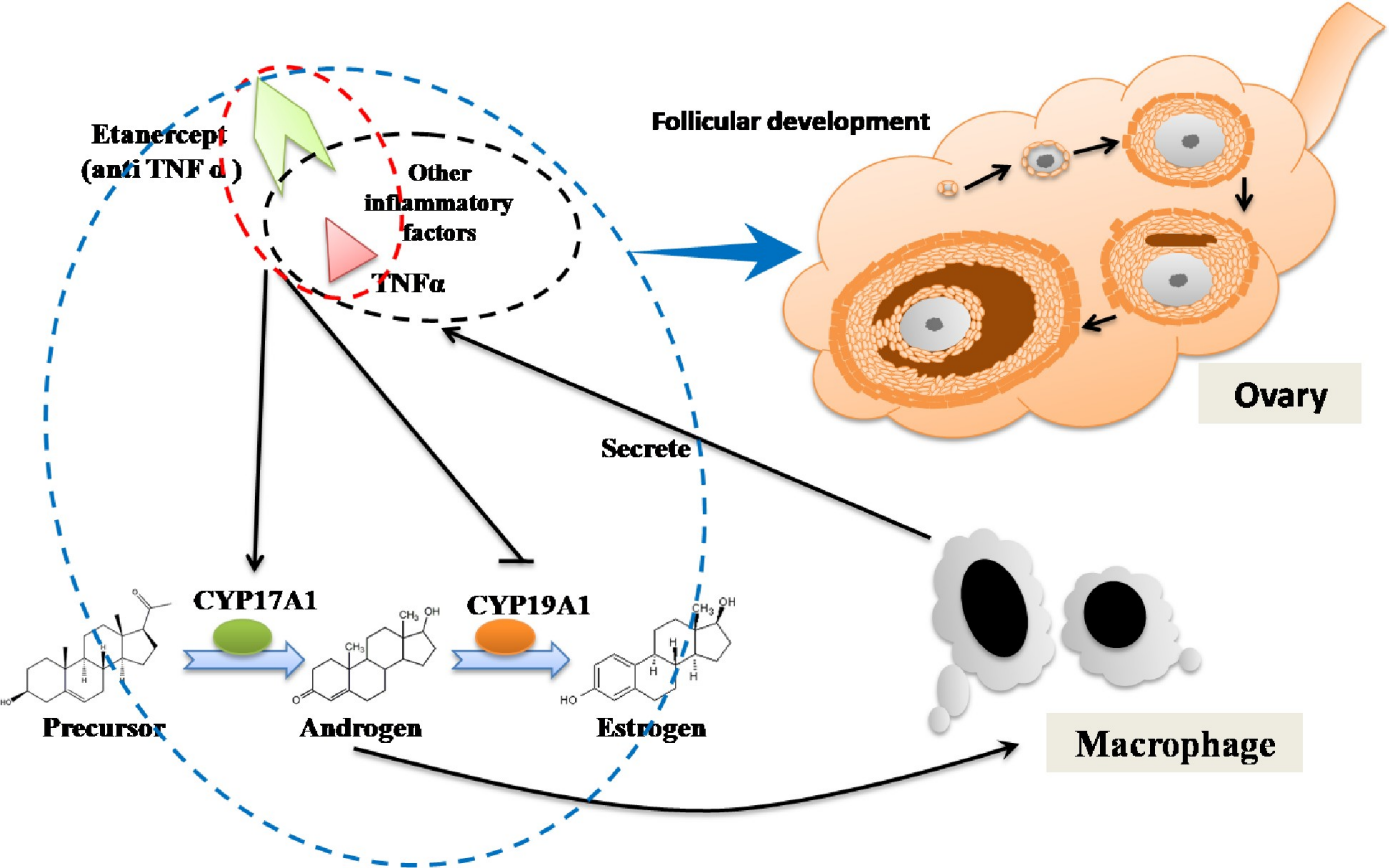
Schematic diagram showing the possible mechanism of anti-TNF-α therapy in polycystic ovary syndrome (PCOS). Persistent exposure to androgen promoted the secretion of TNF-α and other inflammatory factors. TNF-αsignificantly upregulated CYP17A1 and downregulated CYP19A1, the key enzymes involved in the synthesis and conversion of androgens, leading to the increased levels of androgens. Furthermore, etanercept (ETA)-mediated anti-TNF-α therapy in PCOS model significantly alleviated the abnormal hyperandrogenism, lipid recruitment, and follicular development.

Based on the above results, we propose a potential mechanism for the effect of ETA in the PCOS model (Fig 8).

## Discussion

Although chronic inflammation plays a key role in the pathogenesis of polycystic ovary syndrome (PCOS), few investigations on the underlying mechanisms, or on the detailed roles of inflammatory factors in PCOS progression, have been made. TNF-α, a key Th-1 related inflammatory factor, has attracted a lot of attention in a variety of diseases, and numerous studies have confirmed its roles in physiological activities [30–32], which prompted us to investigate the use of TNF-α as a therapeutic agent against the pathogenesis of PCOS.

In the current study, we first tried to apply anti-TNF-α therapy in the treatment of PCOS in a rat model induced by letrozole. Letrozole is a nonsteroidal aromatase inhibitor that inhibits the activity and alters the expression of CYP19A1, which converts androgens to estrogen in the ovary, thereby efficiently decreasing the estrogen/androgen ratio in follicular fluid [21]. Compared with the traditional chemicals used for establishing PCOS rodent models, letrozole can induce comprehensive reproductive and metabolic phenotypes of PCOS. To guarantee a steady and efficient drug release, we implanted 90-day continuous-release pellets subcutaneously in the rats, which released 200 μg letrozole everyday. After 8 weeks of letrozole administration, key features of PCOS, including hyperandrogenism, altered glucose tolerance, chronic inflammation, obesity, and polycystic ovary were detected in the model rats, indicating successful establishment of our model. One thing need to mention is that since letrozole is an aromatase inhibitor, E2 would be expected to decrease. However, our data shows a slight increase instead in in vivo model, and the reason is not fully understand.

ETA, a fusion protein of the TNF receptor and IgG1 Fc, and a widely used clinical TNF-α inhibitor in rheumatoid arthritis, was used in our model to block the effects of TNF-α. ETA showed excellent ability to scavenge TNF-α molecules *in vitro* as well as *in vivo*. Mechanistically, ETA can significantly attenuate the inflammatory condition by decreasing the expression of p65 in the NF-κB signaling pathway induced by lipopolysaccharide (LPS) or testosterone.

Elevated secretion of androgen by the ovary is a key feature of PCOS. Our results indicated that excessive androgen is an important contributor to the chronic inflammation induced during PCOS. TNF-α affects the secretion of androgens, which explains our observation that anti-TNF-α therapy significantly decreased the elevated androgen level in the PCOS model (Fig 8). In terms of the underlying mechanism, several studies have focused on the correlation of TNF-α with androgen and the influence of TNF-α on the androgen receptor (AR) system, whereas fewer investigations have paid attention to the direct effect of TNF-α on androgen synthesis. We used TNF-α to treat ovarian granulosa cells (KGN cell line), and found that it promoted the secretion of androgen, upregulated CYP17A1 (enzyme involved in androgen synthesis) expression, and downregulated CYP19A1 (enzyme that converts androgens to estrogen) expression significantly. In addition, ETA was found to inhibit the inflammatory process. Thus, our results provided new insight into the mechanisms of TNF-α-mediated regulation of androgen synthesis.

Although chronic inflammation has been found to be related to other phenotypes, such as hyperandrogenism, the factors that induce inflammation are still not elucidated. It is widely known that excessive androgen level is a common complication of PCOS and can partially recapitulate the multiple features of PCOS. Dehydroepiandrosterone (DHEA), dihydrotestosterone (DHT), and many other androgens had been previously used to induce the phenotypes of PCOS in a variety of animal models. In the rat model of this study, serum testosterone (T) level was found to be positively correlated with serum TNF-α level. Thus, excessive androgen level was considered as one of the major causes of chronic inflammation. However, it was still not determined whether androgen could cause a significant inflammation in different environments or diseases. Several investigations reported that androgen could alleviate the serious inflammatory condition and protect cells from the damages inflicted by inflammatory factors under certain conditions. Nevertheless, the results of our *in vitro* experiment may partially explain the unknown influences of androgens on inflammation, including short-term exposure of low-dose T that significantly decreased the secretion of TNF-α, transforming mouse macrophage cells (RAW 264.7) to the M2 type, and long-term exposure that aggravated the TNF-α expression and activated the NF-κB inflammatory signaling pathway, transforming mouse macrophage cells to the M1 type. Furthermore, testosterone, but not DHT was used in in vitro model, therefore, our result cannot exclude the possibility that altered estrogen level could also be involved in the regulation, which need further clarification.

Obesity is a classic phenotype of PCOS and is closely related to chronic inflammation. ETA suppressed the excessive lipid recruitment in the PCOS model. It is noteworthy that some relevant clinical studies have reported that anti-TNF-α therapy using ETA in obese patients effectively improved fasting glucose levels and changed the adiponectin composition [33,34]. We believe that in addition to its role in the associations of obesity with hyperandrogenism, chronic inflammation, and other phenotypes, TNF-α is also an important regulator of adipocyte differentiation and lipid recruitment. Adipose tissue plays significant roles in the development of other core symptoms of PCOS, such as ovary phenotype The underlying mechanisms of the communication between adipose tissue and the ovary were extensively investigated. microRNAs (miRNAs) contained within exosomes were speculated as the functional signals, which regulated adipose tissue and other organs [35], and which would be further investigated in our future studies.

In conclusion, ETA could efficiently alleviate the clinical features of PCOS. It efficiently decreased the elevated body weight and serum androgen, TNF-α and MCP-1 levels, and the size of lipid vacuoles, inhibited the expression of pre-adipose differentiation markers, and alleviated the abnormal ovarian condition in PCOS. The results indicated a successful application of anti-TNF-α therapy against PCOS in a rat model, and it is thus speculated that anti-TNF-α therapy could be further explored to be successfully applied in clinical settings or in combination with the conventional treatments in the future.

## Supporting information

S1 File The original Western blot result of the figures used in the manuscript. (RAR)
